# Nanoparticles for treatment of cadmium-contaminated cocoa-growing soils and beans: Performance on metal immobilization and removal

**DOI:** 10.1016/j.heliyon.2024.e40519

**Published:** 2024-12-02

**Authors:** Ambar Oñate, Carina Staël, Darío Bolaños-Guerrón, Carlos Pozo, Daniela Vera, Elena Torres, Carlos Naranjo, Miguel Perugachi, Andrés Loayza, Joffre Pincay, Manuel Carrillo, Luis Cumbal

**Affiliations:** a Centro de Nanociencia y NanotecnologiaUniversidad de las Fuerzas Armadas ESPE, PO BOX 1715-231B, Sangolquí, Ecuador; b Department of Life Sciences and Agriculture, University of the Armed Forces ESPE, PO BOX 1715-231B, Sangolquí, Ecuador; c Department of Energy Sciences and Mechanics, University of the Armed Forces ESPE, PO BOX 1715-231B, Sangolquí, Ecuador; d Soil Laboratories, National Institute of Agricultural Research (INIAP), Mocache, Ecuador; e Department of Earth Sciences and Construction, University of the Armed Forces ESPE, PO BOX 1715-231B, Sangolquí, Ecuador

**Keywords:** Cadmium, Nanoparticles, Cocoa soils, Pollution, Immobilization

## Abstract

Some areas of tropical soils where cocoa grows contain high cadmium (Cd) concentrations. The cocoa plant's need for nutrition causes the reticular system to uptake the toxic metal, translocate it, and accumulate it in roots, stems, and other edible parts such as cocoa beans and shells, threatening the health of cocoa consumers. To cope with this difficulty, different treatments have been applied to cadmium-contaminated soils, but they showed limited success. In this study, we prepared multicomponent nanoparticles (MCNPs) to treat cocoa soils in fixed-bed columns and field tests. Also, MCNPs were mixed with two varieties of cocoa beans, CNN51 and Fino de Aromain, during the fermentation, aiming to capture the cadmium. Our field investigations began by collecting soil samples from three Ecuadorian cocoa-producing farms to determine their physicochemical properties, cadmium, and iron contents. A few weeks later, a home-built prototype was installed in a cocoa plantation to fabricate the nanomaterials using commercial-grade chemicals and rainwater. Scanning Electron Microscope (SEM) images showed MCNPs with an average size of 65.21 nm and the formation of chain-like aggregates. In contrast, MCNPs size was 76.6 nm, measured with Dispersed Light Scattering (DLS). The chemical composition of MCNPs was 90.7 % Fe0 and 1.81 % sulfur (S), analyzed by energy dispersal X-ray (EDX) and confirmed by X-ray diffraction (XRD) spectrometer measurements. Regarding the treatments, for the fixed-bed column tests, 0.28–1.08 L/kg was dosed into the soil, while for field treatments, 250, 500, and 835 mL/min MCNPs were injected in soil areas of 5 m^2^ surrounding each cocoa tree (0.5–4.5 mg MCNPs/kg soil) at depths between 0 and 5 cm. Test results revealed that the procedure applied in the field did not reproduce the metal's immobilization (∼22 %) as in the fixed-bed columns (∼80 %). Moreover, cadmium removal was ∼20 % and ∼75 % after treating CCN51 and Fino de Aroma cocoa beans with 0.112 kg MCNPs/kg in the fermentation on-site and 0.034 kg MCNPs/kg in the laboratory. In this study, the size and chain-like aggregates of MCNPs notably influenced the field treatments as they precluded the filtration of MCNPs downwards. However, immobilizing cadmium could be more effective in soils with higher concentrations of the metal and multicomponent nanoparticles of smaller size, expanding our research scope and offering a practical solution to pollution issues in cocoa-growing regions.

## Highlights

• The home-built and field-operated prototype can supply large-scale multicomponent nanoparticles to immobilize cadmium in on-site applications.
• Preparing in-field nanoparticles using rainwater and commercial-grade chemicals yielded nanoelements with average diameters of 76.6 nm and lengths of a few microns.
• The injection of multicomponent nanoparticles in parcels of cocoa-producing soils and during the fermentation of cocoa beans resulted in 22 % immobilization and 20 % removal of cadmium.

## 1 Introduction

Cocoa (Theobroma cacao), a native bean of the Americas, was a valuable crop in the first South American civilizations. Many researchers report that the plant initially grew in the Amazon and upper Orinoco basins [[1], [2], [3], [4], [5]], but the Mayans and the Aztecs eventually developed techniques to cultivate cocoa successfully [[6], [7], [8]]. Cocoa is produced in humid regions near the Equator, where climate settings are well suited for growing cocoa. Nearly 70 % of the world's cocoa beans come from four West African countries: Ivory Coast, Ghana, Nigeria, and Cameroon. The Ivory Coast and Ghana are the two largest cocoa producers, producing more than 50 % of the world's cocoa [9]. The Ivory Coast alone produced approximately 2.1 million metric tons of cocoa beans in 2021. However, this nation's cocoa production fell 28.5 % in the 2022–2023 crop year due to the El Niño weather phenomenon [10]. In Ecuador, Fino de Aroma and CCN51 cocoa varieties are highly commercial products, and plantations for export are found in several provinces in the Coastal and Eastern regions. In 2022, thousands of tons of cocoa were exported to Europe and the United States, entering millions of dollars into the country's economy [11].

Some soils where cocoa grows contain high cadmium (Cd) concentrations. The cocoa plant's need for nutrition causes the reticular system to uptake the toxic metal, translocate it, and accumulate it in roots, stems, and other edible parts such as cocoa beans and shells [12,13]. The overconsumption of Cd through food can inflict severe harm on vital organs such as the lungs and liver, potentially culminating in cancer and other deadly disorders [14,15]. In response, the European Union and Codex Alimentarius have set limits for cadmium in cocoa and chocolate. The European Union regulations for heavy metals in chocolate have established 0.6 mg/kg as the maximum limit for cadmium in ground cocoa [16]. Meanwhile, the Codex Alimentarius fixed 1.0 mg/kg in chocolate with more than 70 % cocoa [17].

The origin of cadmium in cocoa-growing soils has been related to rock erosion, atmospheric deposition from forest fires, biogenic material, and volcanoes. Like those from South America, soils of volcanic origin contain more Cd than the average concentration reported for natural soils [18]. Accordingly, the high Cd levels in cacao plantations have primarily been attributed to the weathering of parent rocks [18,19]. However, Cd in cocoa-growing soils is also attributed to anthropogenic activities [19,20], mainly by the application of phosphate-based fertilizers [21] and the use of wastewater and irrigation with water rich in cadmium [19,22]. Chavez et al., 2019 specifically report that the metal is of anthropogenic origin in Ecuador due to the watering plants with water rich in cadmium. Likewise, these researchers emphasize that cadmium accumulates at a depth of 15 cm in the soil where cocoa is planted [19]. Thus, distinguishing between geological and anthropogenic sources is essential for managing contaminated soils. On the other hand, evaluating the cadmium bioavailability in soils is more relevant as a cultivation strategy [14], defined as the fraction of soil Cd available to cacao trees for uptake during their lifetime [23]. Bioavailability depends upon various factors that include soil pH, concentrations of metals competing for uptake [e.g., zinc], soil salinity (because of chloride ions), soil organic matter, microbial community, and metal adsorbing iron (Fe) and manganese (Mn) hydrous oxides. Soil pH is considered the most critical component affecting Cd phytoavailability; as soil pH increases, Cd solubility decreases [23]. When soil pH exceeds 6, Cd binds with organic matter, Fe and Mn hydrous oxides, and clay minerals in soil [24,25], reducing its phytoavailability. Microbes perform several mobilization processes, including protonation, chelation, and chemical transformation, whereas sorption or precipitation decreases cadmium availability [19]. Several studies have been conducted on cacao trees in Ecuador [19,26], Honduras [27,28], Peru [29], and Trinidad and Tobago [30,31] examining the relationship between soil and agronomic factors and Cd phytoavailability.

Extensive efforts worldwide have been made to manage soils of cocoa-producing farms contaminated with heavy metals. Modifications with zeolites, humic acids, and selenates have effectively reduced cadmium and lead (Pb) uptake from soil doped with Cd and Pb [32]. Also, Pb, Zn, and Cd were immobilized in soils using biosolids and phosphate rock. The reduced availability of metals achieved with biosolids was better than phosphate rock at alkaline pH. In another study, charcoal was used to reduce high concentrations of soluble Cd and Zn in contaminated soils [33]. Edaphic amendments have been evaluated in cocoa plantations in Ecuador. Mite 2013 treated cocoa-growing soils of three provinces in Ecuador with diverse dosages of vinasse, zeolite, cachaça, charcoal, calcium carbonate, and zinc sulfate, achieving metal decreases between 39 and 58 % [34]. Arguello et al., 2023 reported that soil amendments, such as agricultural limestone, gypsum, and compost, were applied to acidic and pH-neutral soils for two years, achieving different leaf Cd concentrations. Meanwhile, Cd concentration in beans was unaffected by any treatment after 22 months of soil treatment (acid soil) or 30 months (pH-neutral) [35].

The unique properties of nanoparticles, such as their tiny size, high surface-to-volume ratio, and high in situ reactivity [36] have made them very attractive for treatments of sites contaminated with hydrocarbons, organochlorines, pesticides, and heavy metals [37]. In artificially contaminated soils, water-soluble, bioaccessible, and phytoavailable Pb and Cd were reduced with different amounts of nano-hydroxyapatite (nHA) [38]. Moreover, zero-valent iron nanoparticles (nZVI) and cellulosic waste were employed to uptake Pb and Cd in contaminated soil. Both adjustments reduced the phytoavailable fraction of the heavy metals from the contaminated soil [39]. Furthermore, Mallampati et al., 2013 used a nano-Fe/Ca/CaO mixture as an immobilization treatment in soils contaminated with heavy metals (As, Cd, Cr, and Pb), reaching ∼ 95–99 % immobilization of heavy metals [40]. In previous research, we treated artificially heavy-metal-contaminated soil with multicomponent nanoparticles of FeOx/FeS [41]. All heavy metals were immobilized in the soil matrix at values higher than 90 %.

Thus far, a range of amendments at macro and nano levels have been used to reduce the available cadmium in cocoa-growing soils by lab tests and a few on-site treatments [[32], [33], [34], [35],[38], [39], [40], [41]]. Despite the decreased metal content measured in the treated soil, cadmium binding with the inorganic amendments is primarily through electrostatic interaction, precipitation, and formation of complexes, without forming stable solids over time [[37], [38], [39], [40]]. Similarly, using organic amendments does not guarantee permanent immobilization of cadmium, as the organic constituents can undergo biodegradation, thereby releasing the heavy metal [[32], [33], [34], [35]]. On the other hand, treatments using sorbent materials for removing cadmium from cocoa beans in field fermentation have not yet been carried out. Therefore, our efforts aimed to develop a transportable prototype for fabricating MCNPs on a large scale and to use them to treat soil and cocoa beans polluted with cadmium on-site.

## 2 Equipment, materials and methods

### 2.1 The field prototype

#### 2.1.1 Conceptual design of the prototype

**Fig. 1:**
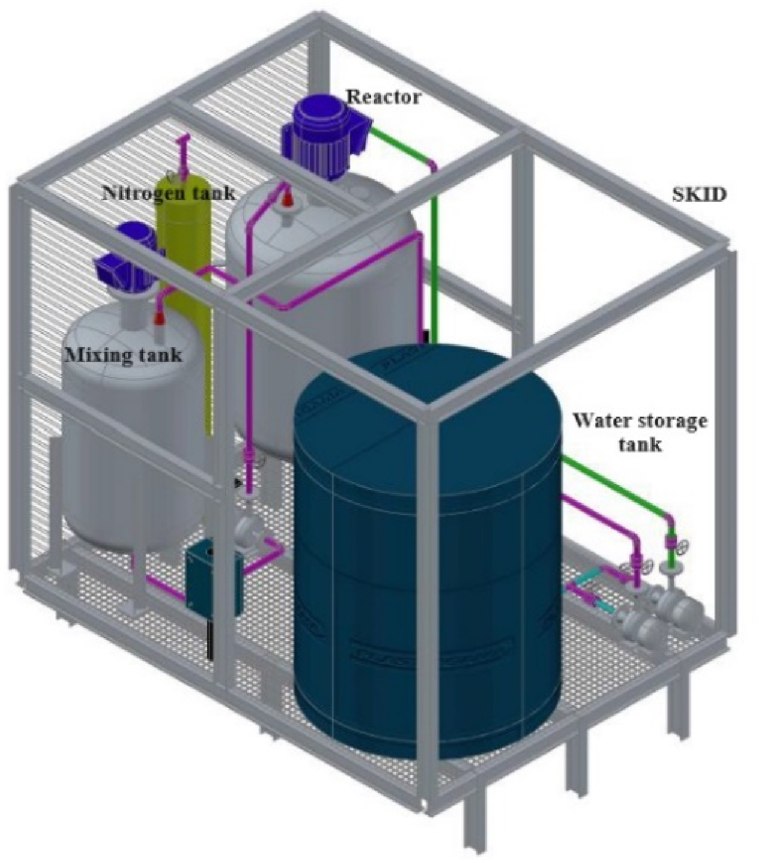
Preliminary scheme of the field prototype.

According to our needs, the field prototype for large-scale nanoparticle fabrication should have a reactor tank, mixing tank, and water storage vessel (Fig. 1). The distinctive features of the tanks are outlined in Table 1. Additionally, the prototype should be designed as a transportable unit for usability across cocoa farms in Ecuador. This adaptability should be carefully met by dimensioning the tanks and related accessories to fit the space of a 4x4 truck's back trunk. The reactor, the vital component of the on-site prototype, should be designed following the best design practices for pressure vessels [[42], [43], [44]], adapted for a volume of 0.5 m^3^. Other features of the reactor are detailed in Table 2. Moreover, an acidic and wet environment will develop inside the tank during the preparation of the nanoparticles, resulting from the dissolution of ferric chloride and other chemical reactants (pH ∼ 2) [45]. Thus, the reactor's body should be planned and built with stainless steel plates [42,43] since it forms a thin layer of chromium oxide that provides corrosion resistance when the steel's surface is exposed to an acidic environment. Furthermore, the design of the reactor's agitation system should match the best practices of the Agitators Design provided by Exxon Mobil in 2001 [46], such as type of agitator, blade designs, spans and angles, type and size of baffles, and operation conditions. To accomplish the reactor's mixing requirements, the agitation mechanism should be conceived with two turbines (PBT: axial flow and RFT: radial flow) positioned concentric to the tank and four baffles incorporated into the vessel walls. After housing all reactor components in a theoretical arrangement, the tank's hydrodynamic behavior should be analyzed through Computational Fluid Dynamics (CFD) [47] simulations for an agitated vessel filled with an aqueous mixture by using the ANSYS FLUENT 17 software in the CAD/CAM Lab of the Universidad de las Fuerzas Armadas ESPE.

**Table 1:** The main characteristics of the field prototype's tanks.

No.	Containers	Agitation	Agitation speed	CFD Simulation	Remarks
1	Main reactor	Yes	400–450 rpm	YES	This container receives all reactant solutions to prepare the nanoparticles. It contains: N_2_, Na_2_SO_3_, FeCl_3_.6H_2_O, CMC, NaBH_4_, and N_2_S.9H_2_O.
2	Mixing tank	Yes	50–100 rpm	NO	This tank handles solutions of NaBH_4_, CMC, and NaOH.
3	Water storage tank	No	No	NO	Only for water storage.

**Table 2:** Data for designing the reactor.

Design parameter	Unit/Type	Value	Remarks
Volume	m^3^	0.5	Lower ellipsoidal tank head
Working fluid	Aqueous phase		Aqueous solutions with chemical reactants
Material	Stainless steel		
Temperature	^o^C	35	
External pressure	atm	1	
Internal pressure	atm	2	
Circulation capacity	m^3^/s	0.2	
Density	kg/m^3^	1000	
Dynamic viscosity	cPs (Pa.s)	10.13 (0.01013)	

#### 2.1.2 Hydrodynamic modeling of the reactor

**Fig. 2:**
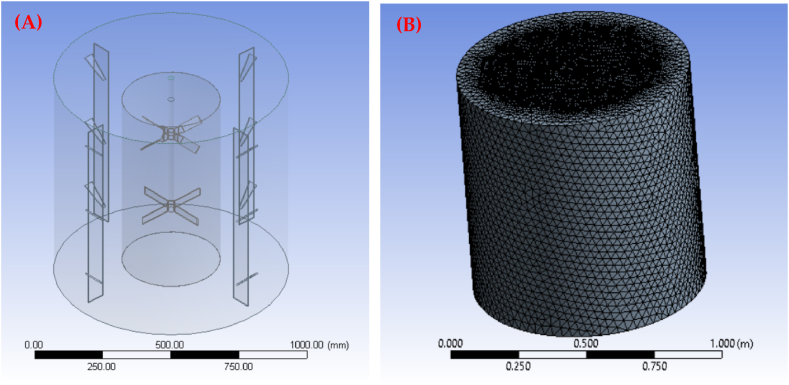
(A) Geometry of the reactor and (B) Computational mesh.

The most challenging part of the reactor's design was generating energy for mixing, shear and elongation stresses, turbulence, and cavitation depending on the speed, viscosity, and other fluid flow properties. This limitation was solved by placing an agitator perpendicular to the cross-section of the reactor's tank and parallel to baffles incorporated on the wall of the tank (Fig. 2a) [48]. To thoroughly evaluate the influence of these components on the fluid's behavior, CFD based on Turbulence Modeling [49] was used. The Reynolds-averaged Navier Stokes (RANS) equation and the kinetic-turbulence (κ–ε) model were used for the numerical modeling of the reactor. In this study, the κ–ε model assumed one-phase fluid (water) and scalable wall functions that allowed an approximate solution of Navier-Stokes equations. The working conditions were defined as: (1) a no-slip condition (v = 0) for all fluid walls when y = 0, (2) activated gravitational conditions because a mixture of aqueous solutions is required, (3) two domains (internal: in the agitation region and external: beyond of the agitator), and (4) a maximum velocity of 420 rpm in the internal zone. Furthermore, the Semi- Implicit Method for Pressure Linked Equations Consistent (SIMPLEC) was used for the solution algorithm that couples the velocity fields with the simulation's pressure fields [50]. This method admits the equations in a transient state being solved with constant relaxation and allows faster convergence of the numerical solution. Discretization patterns of the partial differential equations pressure, momentum, turbulent kinetic energy, and turbulence dissipation rate [51] are as follows: (1) pressure, second order; (2) momentum, second-order forward; (3) turbulent kinetic energy, first-order forward; and (4) turbulent dissipation rate, first-order forward. A computational grid is generated by performing the CFD simulation (Fig. 2b), and the domain is defined by modeling the tank in three dimensions, including the mixing mechanisms. It is crucial to fix the convergence criteria before running the simulation. Those simulations needed 8000–9000 iterations to reach a reliable solution. Additionally, it was essential to verify that the Courant number remained within the range of 0.0–1.0 [52], ensuring the mathematical solution's merging and the accuracy of the simulation. For the application of the turbulence model (k-e) in a defined geometry, it was required to have the mesh close to the vanes with a dimensionless size of y ∼ 5 to attain a satisfactory convergence of the numerical simulation. The element size on the wall was calculated with Eq. (1) [53]:

(1) $$y^{+}=\frac{\rho \times U\times \Delta y}{\mu }$$

where U, ρ, and μ are the fluid's velocity, density, and viscosity, respectively, and Δy is the node size on the wall's impeller.

#### 2.1.3 Construction and operation tests of the field prototype

In the build-up of the field prototype, compliance with its components’ dimensional and technical requirements should be verified. All prototype elements must be assembled inside a reinforced metallic support (SKID) that matches the dimensions of the trunk of a 4x4 truck. After that, a comprehensive verification of the energization system, rotation of the speed reducers installed in tanks, pump operation, and the control panel must be conducted. Ultimately, several MCNPs fabrication tests must be run using the prototype. It must be verified: (1) the water type for preparing the chemical solutions (drinking water, groundwater, and rainwater); (2) the purity grade of chemicals for preparing the aqueous solutions (reagent and commercial); (3) the time required for oxygen depletion inside the reactor; (4) the time needed for the chemical reactions to be completed; and (5) the reactor's rotational velocity for a suitable contact among chemicals. The main goal is to demonstrate that nanoparticles can be manufactured in the field prototype, following a standardized procedure.

### 2.2 Materials and methods

#### 2.2.1 Materials

Reagents were purchased from different commercial vendors. [Sodium carboxymethyl cellulose (CMC, MW: 90000), sodium sulfite (Na_2_SO_3_: >98 % purity), sodium sulfide (Na_2_S.9H_2_O: >99 % purity), Nitric acid (HNO_3_: 70 % purity), perchloric acid (60 % purity), Hydrochloric acid (HCl: 37 % purity), Hydroxylamine hydrochloride (ClH_4_NO: 98 % purity), Hydrogen peroxide (H_2_O_2_: 30 % purity), Sodium acetate (CH_3_COONa: >99 % purity), Magnesium chloride (MgCl_2_: >99 % purity), and Certified Referenced Material Metals in Soil RTC SQC001-30G] vendor: Aldrich; St. Louis, MO, USA. [Ferric chloride (FeCl_3_.6H_2_O: >97 % purity), sodium borohydride (NaBH_4_: >99 % purity), Na_2_S (>99 % purity), and ammonium acetate (CH_3_COONH_4_: >98 % purity)] vendor: Fisher Scientific, USA. Nitrogen gas N_2_(g) (Grade 4: Linde S.A., Ecuador), DI water and rainwater. Commercial-grade chemicals with 60–80 % purity, including sodium sulfide, ferrous sulfate, ferric chloride, and sodium sulfite, were purchased from Espectrocrom, a local dealer company (Quito, Ecuador). Soils and cocoa beans from Ecuadorian cocoa plantations are detailed below.

#### 2.2.2 Methods

##### 2.2.2.1 Synthesis of multicomponent nanoparticles using the field prototype

**Fig. 3:**
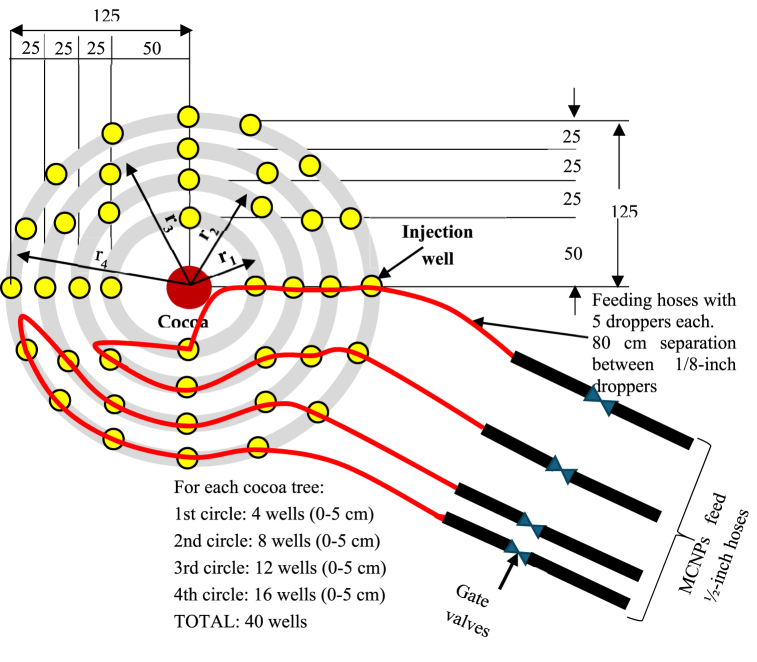
The scheme of a drip doping device for injecting the MCNPs into cocoa-producing soil.

This section only describes the preparation of the MCNPs in the field, even though nanoparticles prepared in the lab were also used in the experimental work. As depicted above (Fig. 1), the prototype for preparing multicomponent nanoparticles holds three tanks: a reactor tank (500 L), a mixing tank (200 L), and a water collector tank (200 L). Before synthesis, the water collector tank is filled with 200 L of rain or groundwater (for physicochemical properties of waters, see Table S1), and from there, water is transferred to the reactor and the mixing tanks (Fig. 3). Both vessels are filled up to the volumes required for synthesis at a 2:1 ratio (reactor:mixer), without exceeding the maximum volume of the reactor. Note that valves A1 and A2 or A1 and A3 are opened according to the tank to be filled with water (Fig. S1). A volume meter measures the liquid level in the reactor. Immediately, synthesis of the nanoparticles starts by adding 25.2 g Na_2_SO_3_ to 120 L of water in the reactor to eliminate the dissolved oxygen (DO), maintaining a speed of 225 rpm for 5 min. The reading on the ORP sensor should be ∼32 mV in the reactor. Next, the gas exhaust valve D4 and the valves D1 and D2 (Fig. S1) in the N_2_ gas line are opened, and 30 L/min is bubbled for 15 min to eliminate the residual OD in the reactor. At the end of this stage, the reading measured by the oxidation-reduction potential (ORP) sensor should be 0.00 mV. Then, 130 g FeCl_3_.6H_2_O, previously pulverized, is added to 120 L of water contained in the reactor and dissolved for 10 min (FeCl_3_.6H_2_O → Fe^3+^ + 3Cl^-^ + 6H_2_O). Without stopping the agitation in the tank, in the first 5 min, an aliquot of approximately 300 mL is taken from the bottom of the reactor to verify if the mixing is achieved. At the end of this step, the ORP value on the sensor should be ∼+522 mV (oxidizing conditions). After that, the mixing tank agitator is turned on and set at 150 rpm to dissolve 60 g NaBH_4_ in 80 L of water (NaBH_4_ → Na^+^ + BH_4_^−^). This step is carried out in the last 5 min of the FeCl_3_.6H_2_O dissolution. Then, the gas exhaust valve D5 is open, and the value in the ORP sensor in the mixing tank should be ∼ −900 mV (reducing conditions). At the same time, valves in the connection line between the mixing tank and the reactor (B1 and B2) are opened. Once the NaBH_4_ dissolution is completed, the solution is transferred to the reactor and mixed with FeCl_3_.6H_2_O components for 5 min at 445 rpm. During the blending, an aliquot is taken from the bottom of the reactor to verify if nanoparticles have been formed by a change in color of the solution, from yellowish to blackish (Fe^3+^ → Fe^0^). At the end of this stage, the value on the ORP sensor should be ∼ −750 mV. After 8 min of FeCl_3_.6H_2_O and NaBH_4_ elements reacting, 1.0 L of 484.3 mM Na_2_S.9H_2_O solution is added to the reactor to cover the Fe^0^ tiny particles with the FeS film, obtaining the multicomponent nanoparticles (Fe/FeS) with a homogeneous shiny black color. Note that to form the FeS cover, it is necessary to keep the solution stirring for 15 min.

##### 2.2.2.2 Characterization of multicomponent nanoparticles

The size distribution of MCNPs in solution was determined using dynamic light scattering (DLS) and the HORIBA software, Version LB-550. The size distribution of the tiny particles was also measured using the field emission gun scanning electron microscope (FEG-SEM, Tescan, Mira3). The operation voltage ranged between 3 and 5 kV. Low-magnification (Å∼2000) micrographs were obtained to measure the size. The elemental composition of the MCNPs was recorded digitally using STEM (Tescan, Mira 3) and equipped with EDX (Bruker Nano GmbH, Quantax). The samples for STEM were prepared by depositing a drop of nanoparticle solution on a carbon-coated copper grid and drying at room temperature. Moreover, the MCNPs' morphology was studied using transmission electron microscope images (TEM, Tecnai G2 Spirit TWIN, FEI, Netherlands). Additionally, to determine MCNPs crystallite composition, XRD studies were carried out using a diffractometer (Empyrean, PANalytical) with a θ-2θ configuration (generator–detector) wherein a copper X-ray tube emitted a wavelength of l = 1.54 Å.

##### 2.2.2.3 Sampling of soils and cocoa beans

Soils from two farms cultivated with cocoa trees, Flavio Alfaro, Manabí province (0°19′23.02″ N −79°48′56.52″ W) were collected. The first was collected near a stream (S001), and the second was atop a hill called Cerro de Oro (S002). Soil samples (S003) were also collected on the Santa Lucia farm (0° 1′ 39.25″ N and 79° 13′ 27.61″ W), La Abundancia, Pichincha province. For sample collection at each farm, plots of 3 × 3 m^2^ were divided into nine cells of 1x1 m^2^. One kilogram of soil was taken from the center of each cell at the surface and depths of 0–15 cm. Then, the nine soil samples were mixed and homogenized, obtaining a composite sample. For physical (texture) and chemical (pH, CEC, cadmium, and other metals) analysis, 1 kg of soil was collected in zip-lock bags. For organic matter analysis, 1 kg of soil was pulled into aluminum foils. For the Santa Lucia farm, four diametral opposite soil samples of 1 kg in circles of r_4_ and r_2_ radii were collected for each tree before and after 1d and 15d of the treatment (for the location of r_2_ and r_4_, see Fig. 3) and then mixed to obtain a composite sample of 2 kg. All soil samples were placed in coolers to keep the temperature between 4 and 5 °C. Moreover, 30 cocoa pods were harvested from the Flavio Alfaro farms, externally washed with distilled water, identified, and transported to the laboratory for further analysis. In addition, 1140 for the Fino de Aroma and 1860 for the CCN51 cocoa pods were collected from the Santa Lucía farm, superficially cleaned with rainwater, and brought to the following process, as detailed in the 2.4.3.3 section.

##### 2.2.2.4 Characterization of cocoa soils and cocoa beans

Before analysis, soil samples were air-dried, ground, and screened through a 2-mm sieve. Subsequently, they were tested using the method ASTM-D2787-11 (2007) [54] to estimate the content of gravel, sand, silt, or clay. The soil's cation exchange capacity was measured following the UNE-EN-ISO-23470 procedure (2018) [55]. ASTM D 2974-00 Standard (2000) [56] and ISO 10390 Standard (2005) [57] were used to measure the organic content and pH, respectively. Atomic absorption spectrometry coupled with a graphite furnace (PerkinElmer, AAnalyst 800) and ICP-EOS (ThermoScientific iCAP 7400) were employed to analyze cadmium and iron [58]. Samples for chemical analysis were extracted from soils using the Tessier sequential extraction method [59] or digested with aqua regia as described below [19]. Concentrations of mg/L and μg/L were converted to mg/kg according to Jankiewicz et al. (2002) [60] Eq. (2).

(2) $$Metal\;\left(mg/kg\right)=\frac{Metal\;concentration\left(mg/L\right)\;x\;Sample\;volume\;\left(mL\right)}{Sample\;weight\;\left(kg\right)\;x\;1000}$$

For cadmium extraction from soil, samples were exposed to sequential extraction tests with a diverse of extractant solutions for determining the content of cadmium in five soil fractions: F1: exchangeable, F2: bound to carbonates, F3: oxidable (bound to Mn and Fe oxides), F4: reducible (bound to organic matter and sulfides), F5: residual (linked to primary and secondary minerals) [59]. The extraction was consecutively performed with an initial weight of 1.0 g of soil following a five-step procedure. Between each step, the resulting solid fraction was carefully washed three times with demineralized water to remove reagent residues and centrifuged to recover the supernatant for use in the next step. Step 1 (cadmium in the exchangeable fraction): 8 mL of 1M NaCH_3_COO adjusted to pH 8.2 were added. The solution was stirred for 1 h at room temperature, then centrifuged for 15 min at 3800 rpm, recovering the supernatant. Step 2 (bound to carbonates): 1.8 mL of 1M NaCH_3_COO adjusted to pH 8 were added to the resulting solid of step 1 and stirred for 5 h at room temperature. Step 3 (bound to iron and manganese oxides): 20 mL of 0.04M ClH_4_NO was added to the residue of step 2 and stirred occasionally, maintaining a temperature of 96 °C for 6 h in a water bath. Step 4 (bound to organic matter and sulfides): 3 mL of 0.02 M NO_3_H and 8 mL of 30 % H_2_O_2_ fixed at pH 2 were added to the residue of Step 3, stirred for 5 h at 85 °C. After that, samples were allowed to cool, and 5 mL of 3.2 M C_2_H_4_O_2_H_3_N was added, centrifuged, and the supernatant was recovered. Step 5 (residual): 8 mL of aqua regia (HNO_3_:4HCl) were added to the previous supernatant, and the content was stirred for 48 h at room temperature. Supernatants recovered from each step were filtered through 0.45 μm and 0.22 μm filters before analytical measurements.

To analyze Cd in the cocoa beans, 0.4 g of ground vegetable material was digested with aqua regia containing 6 mL of 1M HNO_3_ and 2 mL of 1M HClO_4_ at 140 °C for 180 min [19] in a hood. The mixture was stored for 24 h to ensure a complete reaction and a uniform, homogenous mixture. The digested solutions were then diluted to 50 mL with distilled water and filtered through a 0.45 μm membrane filter before Cd analysis.

A standardized procedure was used for the chemical analysis of cadmium [58]. In brief, standard solutions of 0.025, 0.05, 0.5, 1, and 2 mg/L Cd were prepared from a stock solution of 1000 mg/L. A calibration procedure was carried out to standardize the Cd measurements. Then, the soil-extractant fractions and digested solutions were measured for Cd by AAnalyst 800 and ICP-OES [19,58,59]. The limit of detection (LOD) for Cd in soils was fixed at 0.002 mg/kg, and in cocoa beads, 0.01 mg/kg. Quality control for trace and primary element analysis was conducted by employing certificated reference material Metals in Soil RTCSQC001-30G ([CdCl_2_] = 0.025–0.1 %) in each batch. In contrast, the quality control for cadmium in cocoa beads was performed using a locally prepared standard. Each standard was prepared with 1 g of dried cocoa beans, which were then transferred into PTFE vessels. After that, 0.05, 0.25, and 0.5 mL from the 1 mg Cd/L standard solution were spiked to give final spiking concentrations of 50, 250, and 500 μg/kg. The recovery percentages were calculated to determine the matrix effects and measure the method's accuracy [61].

#### 2.2.3 Dosing MCNPs on cocoa-growing soils and cocoa beans

##### 2.2.3.1 In fixed-bed columns

For these tests, fixed-bed columns were packed with 25 g of soils S001 and S002. Then, soils in columns were saturated with 30 mL of distilled water, and subsequently, 7, 14, and 28 mL of 400 mg/L MCNPs were added. Columns remained at rest at room temperature for 24 h to allow the infiltration of MCNPs solution through the soil. For the positive control, 30 mL of 2 % (w/v) agricultural lime was added [62] into the column, and for the negative control, 30 mL of DI water was used. Treated soil was dried in an oven at 40 °C for 24 h. The soil samples were then subjected to sequential extraction and analyzed for cadmium as described above, and the cadmium concentration in the soil samples was determined using Eq. (2).

##### 2.2.3.2 In plots of cocoa-producing soil

Multicomponent nanoparticles prepared in the field were injected in the wells via 1/8-inch droppers at 250, 500, and 835 mL/min flow rates during 2, 5, and 6 min (Table 3). Dosages of MCNPs were injected in soil areas of ∼5 m^2^ (0.5–4.5 mg/kg) surrounding each cocoa tree at depths between 0 and 5 cm. The 40 injection wells were distributed in four circles as depicted in Fig. 3: the first circle radius, r_1_ = 0.5 m (roots of cocoa trees start to grow), four wells; the second circle, r_2_ = 0.75 m, eight wells; the third circle, r_3_ = 1.0 m, 12 wells, and the fourth circle, r_4_ = 1.25 m, 16 wells (Fig. 3). To keep a uniform dropper pressure (30 psi) a closed hydraulic circuit was assembled by connecting two nanoparticle feed lines (see Fig. 3). Soil samples were collected as described in section 2.2.2.3 and pretreated before sequential extraction, as described in section 2.2.2.5. Trees were identified as TR1, TR2, TR3, and TR4 for the soil's sample handling (see Table 6).

**Table 3:** Details of the MCNPs dosing on cocoa-growing soils.

Test	Flow rate (mL/min)	Time (min)	Pressure (psi)	Depth (cm)	Trees/plot
1	250	2	30	0–2	8
2	500	5	30	3–5	4
3	835	6	30	0–2	4

##### 2.2.3.3 In fermentation of cocoa beans

Pods of the Fino de Aroma and CCN51 cocoa varieties were harvested at the farms. Immediately, they were washed with distilled water, dried at ambient temperature, and chopped to free the cocoa beans. Fresh cocoa beans were placed in porous bags, and the mucilage was allowed to drain for 24 h. After that, 100 g of cocoa beans (∼100 beans) of both varieties were placed in a wooden lab fermenter of 10x10 × 10 cm^3^ for the lab fermentation. In the field study, 19 kg of the Fino de Aroma and 31 kg of the CCN51 cocoa beans were placed in a 1.2x1.0 × 0.25 m^3^ field fermenter for on-site treatment (Santa Lucia farm). Temperatures in the fermenters were allowed to rise for 10 h, reaching ∼30 °C. At this stage, the vegetable material was dispersed with MCNPs with doses of 1, 3, and 5 mL/100 g cocoa (Table 4a) (10, 30, and 50 mL/kg) by triplicate for the lab fermentation and 40, 60, and 100 mL/kg for the field fermentation (Table 4b). To keep the temperature inside the fermenters, the beans were covered with banana leaves and jute bags for field fermentation. In contrast, the wooden lab fermenters were placed in an oven at 30 °C.

**Table 4a:** Dosage of MCNPs in the lab fermentation.

Cocoa variety	Dosage (mL MCNPs/100 g cocoa beans)
Fino de Aroma	1
	3
	5
CCN51	1
	3
	5

**Table 4b:** Dosage of MCNPs in the field fermentation.

Dosage (mL MCNPs/kg cocoa beans)	Fino de Aroma cocoa		CCN51 cocoa	
	Cocoa beans weight (kg)	Nanoparticles (mL) dosed per weight	Cocoa beans weight (kg)	Nanoparticles (mL) dosed per weight
40	19	760	31	1240
60	19	1140	31	1860
100	19	1900	31	3100

The fermentation itself lasted four days for the Fino de Aroma variety with the manual turning of beans at 24 and 72 h, and for the CCN51 cocoa, six days with manual revolving at 24, 72, 96, and 120 h. At the end of the process, both cocoa bean varieties were dried in a lab drier and canopy in the field. From the sixth day of drying, the percentage of humidity was monitored with Aqua-Boy equipment. The optimal humidity rate (7 %) was reached on the eighth day for both treatments. Next, cocoa beans were randomly sampled and distributed in triplicate samples. Ultimately, on fermented beads, the cotyledon was separated from the shell and ground in an IKA-type fabric mill for subsequent digestion and quantification of cadmium by AAnalyst 800.

#### 2.2.4 Statistical analysis

The data obtained from the cadmium immobilization in the soil of fixed-bed column tests and field treatments and the metal removal from cocoa beads in lab fermentation was analyzed using the OriginPro package. The Shapiro-Wilk normality test was conducted to investigate the variance (ANOVA) in Cd data in lab fermentation of cocoa beans and treatments in fixed-bed columns. For Cd data resulting from the field treatments, parametric tests (Shapiro-Wilk) were performed for fractions 3, 4, and 5 and non-parametric (Kruskal-Wallis) for fractions 1 and 2. Then, the Tukey test (for parametric) and the Dunn test (for non-parametric) were conducted to compare the means in each period. In all hypothesis tests, a significant level of 5 % (α = 0.05) was used as the standard, and a p-value below 0.05 was considered a statistically significant value.

## 3 Results

### 3.1 The on-site prototype

**Fig. 4:**
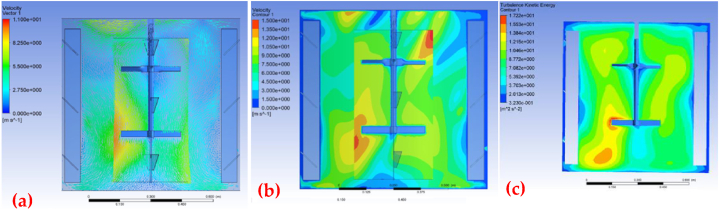
**S**imulation of the mixing in the reactor at t = 60 s. **(a)** Velocity vectors, (b) Velocity contours, (c) Turbulent kinetic energy.

**Fig. 5:**
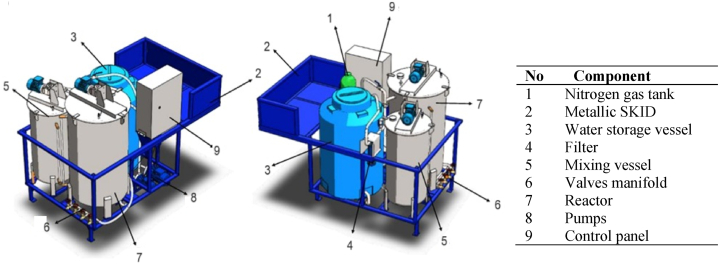
Components of the field prototype.

**Fig. 6:**
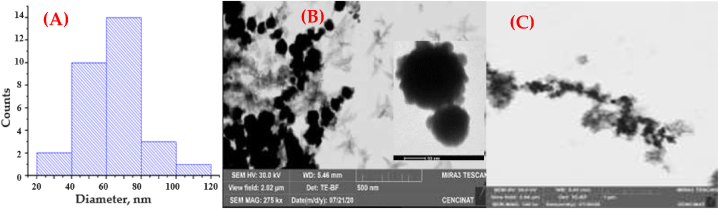
MCNPs prepared with the in-site prototype: (a) Size distribution with DLS (diameter = 76.6 ± 27.11 nm), (b) Morphology of spherical nanoparticles imaged with SEM (diameter = 65.21 ± 16.12 nm), and (c) SEM of agglomerated nanoparticles forming a chain like structure.

Based on the CFD simulation outcomes, the impeller-blade configuration becomes an appropriate mechanically agitated mixer. The velocity vector field in Fig. 4a shows that this mechanism achieves good mixing throughout the reactor. After 60 s of agitation, many vorticities are observed inside the reactor, confirming an efficient fluid mixing. Fig. 4b further validates the reactor's mixing effectiveness, showing a well-homogenized mixture with few zero-velocity contours. It is also observed that the flow is developed from the internal domain towards the entire container, showing consistent values over time. Additionally, the existence of turbulent kinetic energy (TKE) in the CFD simulation (Fig. 6c) confirms an adequate mixing of the fluid [63]. Turbulent energy values ranging between 0.32 and 17 m^2^/s^2^ are achieved, and the calculated fluctuation's average velocities vary between 0.6 and 4.5 m/s. Our simulations align with those reported by Torotwa and Changying, 2018. They found that the impeller-blade configuration influences the performance of agitated mixers, suggesting that the agitation systems in stirred tanks are the mechanisms that control the mixing [64]. From the construction perspective, Fig. 5 shows the prototype with its main components: reactor, water storage vessel, and mixing vessel, as well as pumps, gear motors, electric generator, control panel, filter, control panel, container for transporting tools, and nitrogen bottle. All of them are assembled in the SKID that fits in the trunk of a 4x4 truck. Functioning tests demonstrated that the prototype is quickly energized by energy from the public network or with a portable diesel generator. Speed reducers in vessels, piping valves, flow line pumps, and a control panel worked perfectly. Also, it was proven that the prototype is compact and stable equipment that can be safely transported to Ecuador's cocoa-producing farms. It was raised and lowered from the truck's trunk with the assistance of a 2-ton lift. Results of the MCNPs synthesis are provided in the sections below.

### 3.2 Nanoparticles characterization

Characterization of MCNPs prepared on-site provided the following data: The pH of the nanoparticle solution was 8 due to the usage of NaBH_4_ as the reducing agent. Nanoparticles had an average size of 76.6 nm measured with the DLS (Fig. 6a) and with SEM 65.21 nm (Fig. 6b).

**Fig. 7:**
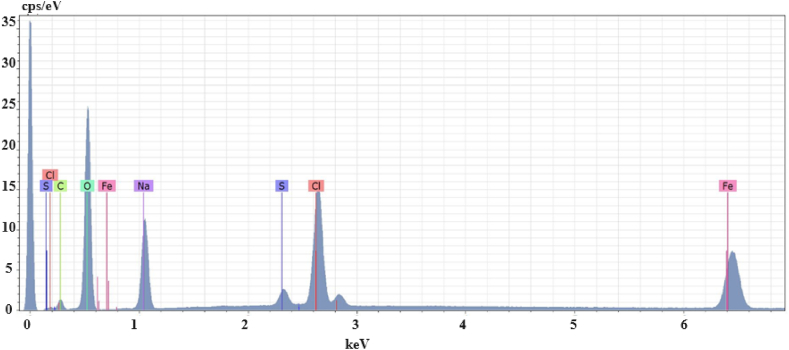
The chemical composition of the MCNPs prepared using the field prototype.

The discrepancy in size is because of the methodology used for measurements. With the DLS, nanoparticles are in solution and thus surrounded by water molecules, whereas, for SEM measurement, the water film is evaporated. The micrographs of the MCNPs showed homogeneity in shape and size. SEM images also showed a filament-like morphology of MCNPs (Fig. 6b and Fig. S2b). Additionally, the presence of FeS precipitates surrounding the surface of the nanoparticles (Fig. 6b) yielded roughness, confirmed by the atomic force microscope (AFM) image. It showed an almost homogeneous roughness on the surface of the nanoparticles (Fig. S3). Moreover, the chemical composition of the MCNPs obtained by the EDX technique is shown in Fig. 7. The iron (Fe^0^) content is 90.7 %, and the sulfur (S) is 1.81 %, corroborated by a homogeneous content of iron and sulfur mapped with the SEM (Fig. S4). Furthermore, the XRD spectrum confirms the presence of elemental iron in the nanoparticles (Fig. S5). The peaks at 2θ: 44.79°, 65.11° and 82° are related to elemental iron [65,66], and the peaks at 2θ: 30° and 35.8° with pyrite (FeS) [67]. Conversely, MCNPs prepared in the lab for fixed-bed column tests measured an average diameter of 17.2 nm (Fig. S2a). The chemical composition by EDX is Fe = 61.50 %, S = 2.48 %, C = 5.91 % and O = 30.11 %. Notably, the latter two elements (C and O) indicate the formation of a fine organic layer bound to the surface of the nanoparticles. This result is due to polyphenols in the vegetable extract used as a secondary stabilizing agent [68]. The XRD spectrum shows a peak for elemental iron (α-Fe^0^) at 44.69° and a peak at 35.44° assigned to the crystallographic plane 110 (JCPDS Nº 33–0664) of the iron oxides (Fig. S2c). However, sulfur-containing compounds, such as FeS, cannot be observed in the spectrum because of their low concentration or low degree of crystallinity [41,69].

The difference in MCNPs’ size between both synthesis procedures can be associated with the purity of the materials and water quality used. The infield-prepared large nanoparticles were grown using commercial-grade chemicals and rainwater. In contrast, the lab-prepared small nanoparticles were synthesized using reactive-grade chemicals and deionized water. However, in terms of nanoparticle final components, our results align with those reported by Stäel & Cumbal 2016 and Kim et al., 2011 [70,71].

### 3.3 Cocoa-producing soil properties

Table 5 summarizes the main physicochemical properties of soils used in this study. Textural composition for Flavio Alfaro soils, S001 with (20 % sand +80 % fines and S002 with 14 % sand +86 % fines and soil of Santa Lucia farm, S003 with 12 % sand +88 % fines, correspond to clayey type [54]. The three soils have fair organic matter content (S001 = 4.2, S002 = 6.61, and S003 = 15.06 %) within those measured in humid Andisol soils. Organic matter contents as high as 20 % can be found in Andisols in warm and humid climates [72]. Moreover, the cation exchange capacity (CEC) for soils S001, S002, and S003 is 8, 9.3, and 13.99 meq/100g, respectively, similar to those reported by Kaiser et al., 2008. Authors found CEC values in the 7.3–12.9 meq/100g range for soils with 11.5 g/kg of organic matter at pH∼6 and established that CEC is a pH-dependent property [73]. Furthermore, hydrogen potential (pH) is slightly acidic (pH = 5.08–6.33) and suitable for cocoa cultivation (pH = 5.6–7.2) [74]. Additionally, the iron concentration of the three soils is slightly high (10,853–19,081 mg/kg); the iron comes from the volcanic origin media (Andisols) [75]. Finally, the cadmium concentration for soils from Flavio Alfaro is 2.32 and 2.27 mg/kg for S001 and S002, respectively, and for Santa Lucia soil, 0.45 mg/kg. The concentration of cadmium in all tested soils is above the mean Cd concentration in non-polluted European agricultural soils (0.3 mg/kg) and median soil Cd in the USA (0.2 mg Cd/kg) [76].

**Table 5:** Main physicochemical properties of soils from three cocoa-producing farms.

Property	Units	Soil		
		Flavio Alfaro S001	Flavio Alfaro S002	La Abundancia S003
Sand	%	20	14	12
Fine particles	%	80	86	88
Organic matter	%	4.20	6.10	15.06
pH		6.33	6.21	5.08
CEC	meq/100 g soil	8.00	9.30	13.99
Fe in soil	mg/kg	10853	11019	19081
Cd in soil	mg/kg	2.32	2.17	0.45

### 3.4 Immobilization of cadmium in soils treated with MCNPs

**Fig. 8:**
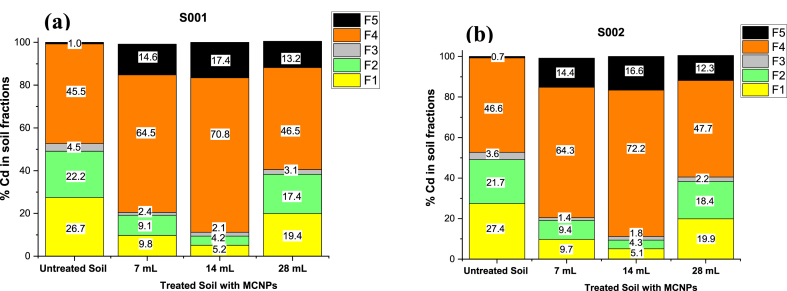
Cadmium distribution in Flavio Alfaro soil fractions after treatment with 7, 14, and 28 mL of 400 mg/L MCNPs. (a) Sample S001 and (b) Sample S002.

**Fig. 9:**
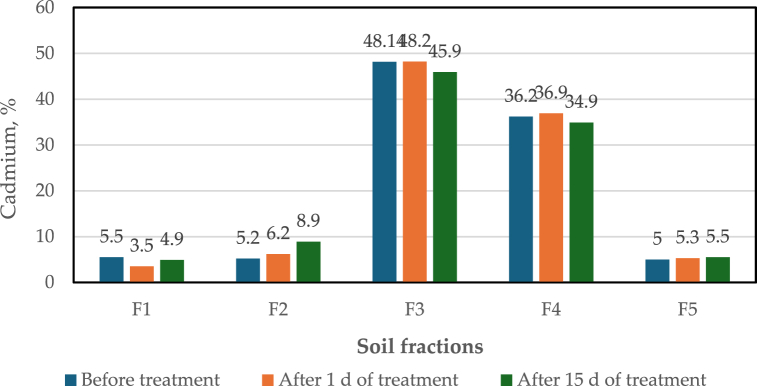
Distribution of cadmium in soil fractions at three different treatment times.

Fig. 8a and b shows high percentages of available cadmium immobilization in both soils when dosing the columns with 14 mL of MCNPs. In sample S001, cadmium decreased in the exchangeable (F1) and carbonated (F2) fractions from 26.7 % to 5.2 % (80.5 %) and from 22.2 % to 4.2 % (∼81 %), respectively (Fig. 8a). Similarly, with soil S002, cadmium decreased from 27.4 % to 5.1 % (∼81 %) in F1 fraction and from 21.7 % to 4.3 % (80.2 %) in F2 fraction (Fig. 8b). On the contrary, in both soils, cadmium increased in the fraction bound to organic matter and sulfides (F4) (35.7 % for S001 and 35.5 % for S002), and in the residual fraction (F5) (94.4 % for S001 and 95.8 % for S002) (Fig. 8a and b). These findings suggest that the mobility and bioavailability of Cd in the soils are significantly reduced. Likewise, when using 7 mL of MCNPs in the treatment, a decrease of cadmium in F1 and F2 fractions (63.9 % for S001 and 64.6 % for S002) and (59 % for S001 and 56.7 % for S002), respectively. Moreover, there was an increase of Cd in the F5 fraction for both soil samples (S001 = 93.1 % and S002 = 95.1 %) (Fig. 8a) and a slight rise in the F4 fraction (S001 = 1.7 % and S002 = 2.3 %) (Fig. 8b). It is evident that when fewer MCNPs are injected into the column, less cadmium is immobilized. The analysis of variance (ANOVA) for the exchangeable fraction (F1) for both soils results in a p-value of <0.0001 (Table S2 and Table S3), less than the significance level α = 0.05. Thus, cadmium immobilization depends on the volume of the injected nanoparticles as it drives the metal transfer to F4 and F5 stable fractions. However, when 28 mL of MCNPs were added to the fixed-bed columns, the immobilization of cadmium was poor (Fig. 8a and b) because of nanoparticles agglomeration on the top of the column soil, thus allowing filtration of only a small MCNPs portion. Furthermore, Table 6 summarizes values of the cadmium extracted from the soil fractions for the on-site tests for the best-balanced flow rate (835 mL/min of MCNPs). Fig. 9 shows that after 24 h of treatment, the average metal concentration in the F1 fraction decreased from 5.48 to 3.50 % (37 % of Cd immobilization). In contrast, cadmium slightly increased in the F5 fraction from 5.02 to 5.27 % and in the fraction F2

**Table 6:** Cadmium concentrations in five soil fractions at different treatment times per tree.

Soil Fract	TR1 BT	TR1 1D	TR1 15D	TR2 BT	TR2 1D	TR2 15D	TR3 BT	TR3 1D	TR3 15D	TR4 BT	TR4 1D	TR4 15D
Cadmium (mg/kg)												
F1	0.023	0.014	0.014	0.024	0.015	0.031	0.023	0.014	0.017	0.021	0.014	0.021
F2	0.017	0.024	0.025	0.024	0.026	0.057	0.023	0.028	0.031	0.023	0.023	0.039
F3	0.181	0.206	0.218	0.214	0.195	0.180	0.206	0.209	0.201	0.195	0.177	0.189
F4	0.149	0.150	0.153	0.154	0.151	0.150	0.148	0.154	0.146	0.150	0.148	0.149
F5	0.021	0.021	0.023	0.020	0.022	0.023	0.021	0.022	0.024	0.021	0.021	0.023

**TR1**: Tree 1, **TR2**: tree 2, **TR3**: tree 3, **TR4**: tree 4 (one plot of 12 m^2^).

**BT**: before treatment; **1D**: one day after treatment; **15D**: fifteen days after treatment.

**F1**: exchangeable cadmium, **F2**: cadmium bound to carbonates, **F3**: cadmium bound to metallic oxides, **F4**: Cadmium linked to organic matter, **F5**: cadmium in the residual fraction.

from 5.2 to 6.18 %. Overall, cadmium immobilization in the soil was ∼22 %. However, the percentage of metal immobilization in the same plot after 15 days of treatment decreased to 12 %, a result that needs further investigation. Cadmium also increased in fractions F1 and F2 to 4.90 and 8.86 %, respectively. The statistical analysis was conducted using the Cd data of each soil fraction, as shown in Table 6. The parametric (one-way phase ANOVA) tests for F3, F4, and F5 and the non-parametric for F1 and F2 resulted in a significant difference between the original state (soil without treatment) and after treatment (α = 0.05). Thus, the Tukey test (for parametric tests) for F3, F4, and F5 and the Dunn test (for non-parametric tests) for F1 and F2 were run to compare the means in each period. These schemes show that in F1, there is a significant decrease in Cd after 1 day of treatment with MCNPs, and it remains almost the same 15 days later. In contrast, in F5, Cd increases significantly after treatment (Fig. S6). Moreover, F2, F3, and F4 have no change in the Cd content. Our results align with Guha et al. (2020) [77]. The authors reported that the modulation of the transport and immobilization of Cd by iron nanoparticles drives the metal transfer from F1 to F5. Moreover, the slight increase in the concentration of Cd in F1 after 15 days of treatment could be linked to different factors, such as the oxidation of the nanoparticles or the release of the Cd accumulated in cocoa litter [78].

### 3.5 Removal of cadmium from cocoa beans using MCNPs during the fermentation

For the fermentation tests conducted in the laboratory, cocoa beans collected in Manabi province had cadmium concentrations of 1.7 and 1.3 mg/kg for the Fino de Aroma and CCN51, respectively, above the permissible level of 0.6 mg/kg [16,17]. Table 7 shows that 3 mL of MCNPs per 100g of beans (30 mL/kg) was the best treatment for both cocoa bean varieties. The Cd removal was 80.59 and 69.23 % for the Fino de Aroma and CCN51, respectively. In comparison, cadmium removal decreased when applying 1 and 5 mL of MCNPs. This response is due to the small amount of MCNPs interacting with the cocoa beans during fermentation for a 1 mL dosage. In contrast, the 5 mL caused agglomeration of MCNPs on the surface of the beans. The analysis of variance (ANOVA) for Cd concentration after lab fermentation of the Fino de Aroma cocoa variety reveals that the Volume of MCNPs (Concentration) has a p_value of 0.000373 (Table S4). Meanwhile, for CCN51, the p_value is 0.011 (Table S5). Both p_values are less than the significance level α = 0.05. Thus, the volume of MCNPs significantly affects the removal of cadmium during fermentation. Statistically, the 3 mL of MCNPs is the best treatment as it produces a higher removal of Cd during cocoa fermentation.

**Table 7:** Cadmium removed from cocoa beans in the laboratory fermentation.

Cocoa variety	Nanoparticles dosage (mL)	Cadmium before treatment (mg/kg)	Cadmium after treatment (mg/kg)	Cadmium removal (%)
Fino de Aroma	1		0.40	76.47
	3	1.7	0.33	80.59
	5		0.56	67.06
CCN51	1		0.70	46.15
	3	1.3	0.40	69.23
	5		0.50	61.54

On the other hand, in the fermentation study conducted in the field with cocoa beans from the Santa Lucia farm, Table 8 shows that Cd removal using 100 mL/kg of cocoa beans was 20 % for the Fino de Aroma variety and for the CCN51.

**Table 8:** Cadmium removed from cocoa beans in the field fermentation.

Test	Cocoa variety	Dosage (mL NPs/kg cocoa beans)	Cadmium (mg/kg)	Average removal (%)	Cocoa variety	Dosage (mL NPs/kg cocoa beans)	Cadmium (mg/kg)	Average removal (%)
1	Fino de Aroma	40	0.41	11	CCN51	40	0.40	**8**
2		40	0.40			40	0.42	
3		40	0.40			40	0.44	
1		60	0.38	15		60	0.38	**15**
2		60	0.38			60	0.39	
3		60	0.40			60	0.39	
1		100	0.37	20		100	0.37	**19**
2		100	0.37			100	0.37	
3		100	0.36			100	0.36	

19 %. Lower percentages of Cd removal were obtained by applying minor dosages (40 and 60 mL/kg). Thus, the volume of MCNPs used in the treatments is also critical in removing Cd in the field fermentation. Despite these limitations, the quality of the fermented cocoa beans after treatments with MCNPs produced a seed index between 1.15 and 1.49, a humidity of 5.5–7 %, and pH values in the range of 5.23–5.57, meeting the local standards [79]. Moreover, 91.33 and 93.33 % of good fermentation resulted in the Fino de Aroma and CCN51 above 75 % standard of good fermentation [80].

### 3.6 Comparison with other cocoa soil treatments

The results achieved in this investigation are compared with other studies, as shown in Table 9. First, our test results for Cd immobilization in soils conducted in the lab differ from those in the field. This is due to the size of the MCNPs and the environmental and operational conditions. The results achieved in this investigation are then compared with studies of available Cd reduction in soils, as shown in Table 9. First, our test results for Cd immobilization in soils conducted in the lab differ from those in the field. This is due to the size of the MCNPs and the environmental and operational conditions. Second, the results of reducing cadmium by applying MCNPs in soils from the Santa Lucía farm were different from treatments performed with other amendments, such as biochar [81], lime [82], and compost [83]. Also, the experimental conditions of tests were diverse, such as soil type, soil pH, Cd content in untreated soil, dosage rates of the amendment, and testing times. However, this study includes the following advantages: (1) We conceived and built a field prototype for large-scale nanoparticle fabrication, which can be considered a technological innovation. (2) As proven in previous research, multicomponent nanoparticles linked to the available cadmium form stable solid compounds, such as CdS, CdO, and (FeOH)_2_Cd, that are not easily dissolved by average rainfall [41]. In contrast, the cadmium bound to the organic materials (biochar and compost) is released due to the digestion of organic amendments by soil bacteria. Also, lime dosage should be managed carefully to avoid increasing soil pH; otherwise, the Cd reduction capability will be affected because a positive surface charge rejects Cd^2+^ ions. (3) MCNPs dosed into soils in fixed-bed column tests reduced available cadmium by 81 %, a higher percentage than the other treatments (biochar: 71 %, lime: 30–40 %, compost: 19–30 %). However, the metal's immobilization decreased due to the increase in the size of the MCNPs and the environmental conditions on the soils of the Santa Lucia farm. (4) Due to nanoparticle reactivity, treatments with MCNPs are kinetically faster (pseudo-second-order kinetics) [41] than those using the other amendments. After 1 and 15 d, our particles were evaluated for cadmium immobilization in cocoa soils, resulting in moderate metal retention. The other methods needed more time (60–180 d). Therefore, our process can be applied to extensive areas of cocoa-cultivated soil. However, our process needs higher sorbent material (MCNPs) rates for in-field soil treatments, making it more expensive. 5) Our MCNPs can be added during the fermentation of the cocoa beans in the lab and the field fermenters. For Fino de Aroma and CCN51 cocoa varieties, nanoparticles yielded Cd removals of 20 % for in-field and ∼75 % in the lab tests without affecting the quality of fermented beans.

**Table 9:** Comparison of cadmium content in soil and cocoa parts after diverse treatments.

Author	López et al. 2022 [81]	Ramathal et al., 2019 [82]	Bagkaie, 2018 [83]	This study		
Amendment	Biochar	Lime	Compost	MCNPs		
Study site	Lab	Field	Field	Lab	Field	
Soil type	Loamy	Salty loam	Loamy	Clayey	Clayey	
Soil pH	Acidic = 5.52 Alkaline = 7.30	4.90	7.00	6.21–6.33	5.08	
Cd in virgin soil	0.6 ± 0.1 mg/kg	0.32 mg/kg	20 mg/kg	2.17–2.32 mg/kg	0.45 mg/kg	
Amendment rate for soils	1–2%	0–0.6 kg/m^2^	0–3 kg/m^2^	0.31–1.13 kg/kg soil	0.5–4.5 kg/m^2^	
Testing time for soils	130 d	180d	60d	24h	24h	15d
Cd reduction in soil	71 %	30–40 %	19–30 %	∼81 %	22 %	12 %
Cocoa type	CCN51	–	–	CCN51 Fino de Aroma	CCN51 Fino de Aroma	
Amendment rate for cocoa parts				0.01–0.056 kg/kg	0.04–0.112 kg/kg	
Testing time for cocoa parts	130 d	180d	60d	4–6d		
Cd reduction in cocoa parts	48 % (leaves)	20–37 % (leaves)	66 % (shoots)	46.2–80.6 % (beans)	19–20 % (beans)	

## 4 Discussion

The home-built reactor operated in the field yielded MCNPs with an average diameter of 76.6 nm (Fig. 6a) and chains of spherical nanoparticles measuring a few micrometers long (Fig. 6c). They are larger than those prepared in the laboratory (17.2 nm). The growth of such nanoparticles could be influenced by the interaction between rainwater pollutants collected at the Santa Lucia farm (see Table S1) and commercial-grade chemicals (∼60 % purity) during synthesis, producing nanoparticles and tiny residues due to incomplete chemical reactions. Miniature artifacts dissolve and then redeposit onto larger particles, usually increasing their size [84]. Despite both types of MCNPs containing the same components, Fe^0^ in the core and FeS in the shell, immobilization of the cadmium in column tests conducted on screened soils dosed with MCNPs of 17.2 nm yielded better results than those carried out in the field with 76.6 nm MCNPs. Overall, the MCNPs treated cocoa-tree soils showed a vast decrease in cadmium concentration (37–80 %) in the exchangeable fraction (F1), a reduction (19–80 %) in the metal bound to carbonates (F2), and an increase in Cd (5.7–35 %) in the reducible (F4) and (5–95 %) residual (F5) fractions. The drop of Cd concentrations in the F1 and F2 fractions is caused by the capture of the weak binding metal by Fe^0^ and FeS reacting groups of the MCNPs. Conversely, the F5 increase suggests a transformation of the zero-valent iron into oxidized forms such as goethite (α-FeOOH) or maghemite (γ-Fe_2_O_3_) that strongly immobilize cadmium by the formation of inner-sphere complexes, such as (FeO)_2_Cd, FeOCd^+^, and FeOHCd^2+^ and CdO precipitates [[85], [86], [87]]. Furthermore, the increase of cadmium in the reducible fraction (F4) is caused by the rapid interaction of FeS with the metal, forming CdS precipitates [88,89], a reaction accelerated by the CdS solubility product (Ksp_,CdS_ = ∼10^−29^) [90]. Our past kinetics study supports these findings; 99.5 % of cadmium was removed from 10 mL of the artificially contaminated water in 5 min, employing 1 mL of MCPNs with an average size of 9.5 nm, corresponding to pseudo-second-order kinetics [41]. Additionally, the precipitates of FeS deposited over the Fe^0^ core of the nanoparticles generated a rough finish—the tiny valleys and peaks of the roughness supply MCNPs with an extra surface area. In an AFM image (Fig. S3), it is observed that roughness and the surficial area increase when more FeS precursor (591 mg/L Na_2_S.9H_2_O) is added to the MCNP synthesis. Kim et al., 2011 reported that when dithionite (FeS precursor) concentration was increased from 1 to 4 g, the average surface area increased from 25.3 to 42.2 m^2^/g [69]. On the other hand, MCNPs applied in the fermentation of cocoa beans yielded lesser cadmium elimination in field treatments (∼20 % for both cocoa varieties) than that obtained in the laboratory fermenter (Fino de Aroma: 80 % and CCN51: 69 %). Like in soil treatments, larger MCNPs (76.6 nm) partially diffused across the pore size of cocoa beans (22.6 nm) [91]; thus, fewer nanoparticles would interact with cadmium, decreasing its removal. The large size (76.6 nm) and chain-like aggregates of the MCNPs prepared in the field prototype played an essential role in cadmium adsorption in on-site tests as they hindered nanoparticle infiltration in Santa Lucia's soil and across the cocoa beans. Also, the cadmium content in the cocoa beans for laboratory fermentation was higher (Fino de Aroma = 1.7 mg/kg and CCN51 = 1.3 mg/kg) than in cocoa beans from Santa Lucia farm (Fino de Aroma = 0,46 mg/kg and CCN51 = 0.45 mg/kg). Consequently, cadmium removal in laboratory fermentation increases due to the relatively high metal concentration of cocoa beans.

On the other hand, the soils from Flavio Alfaro (S001 and S002) and Santa Lucia farm (S03) treated with MCNPs match the clayey category [54]. These soils are ideal for cocoa-growing systems as they offer moderate to adequate water infiltration, and only a minor percentage needs better drainage [92]. However, they influenced the performance of on-site treatments in diverse ways. For instance, the microporosity of the clay (diameter ranges between 1 and 100 nm) [93] can hinder the infiltration of the injected MCNPs (sizes of 166 ± 111 nm), leading to the formation of pools of nanoparticles on the top of the on-site treated soils. The three soils hold moderate organic matter content (4.2–15.06 %) and, thus, can accumulate cadmium on the reducible fraction, F4 = 46 % for S001 and S002 and ∼38 % for S003. The CEC for the treated soils ranges from 8 to 13.99 meq/100 m. However, the exchangeable cadmium (F1) is ∼27 % for S001 and S002 soils and ∼6 % for S003 when contacted with MCNPs. So, when exchangeable Cd is released, it mainly binds to hydrous-oxides (F3) or ferrous sulfide (F4). On the other hand, the iron concentration of the three soils is slightly high (10,853–19,081 mg/kg). Andisols, like most of Ecuador's soils, are iron suppliers as they contain a substantial amount of hydrous ferric oxyhydroxide minerals (i.e., ferrihydrite) [75], which potentially bound cadmium thus increasing Cd in the oxidable (F3) or in the residual (F5) fractions. In addition, the textural composition of the three soils (80–88 % fines and 12–20 % sand) allows less weathering and drainage; thus, iron is easily oxidized [94]. Eventually, iron oxides bind heavy metals like cadmium and accumulate in the F3 (∼50 %) and F5 (∼5 %) fractions for S003 soil. However, this tendency for cadmium accumulation in F3 (∼4 %) and F5 (∼1 %) is not observed in S001 and S002 soils, although iron content is above 10,000 mg/kg, probably because iron forms Fe-organic matter complexes [95,96] preventing interactions with Cd. Furthermore, the hydrogen potential (pH) of soils is slightly acidic (pH = 5.08–6.33); under this pH condition, Cd is relatively free to move because it is replaced by H^+^ ions (2.51189x10^−6^–4.67735x10^−7^ M) and, to some extent, travels across the soil's pores for interaction with reactive groups of MCNPs or soils. This transfer of Cd under slightly acidic conditions might pose a potential risk in the context of soil treatment.

The moderate immobilization of cadmium obtained in the field treatments (S003 soil) suggests that environmental and operational processes played a role in the metal retention. (**1**) In the field study, intensive rains fell (annual precipitation ∼2127 mm) over the Santa Lucia farm for several hours (18 rainy days in December 2021), reaching an average of up to 197 mm in the month [97], which may have stimulated the release rate of Cd from stable soil fractions (F3, F4, and F5). Previous investigations revealed that heavy metals, including cadmium, were immobilized in soils treated with MCNPs in fixed-bed columns. Treated soils were leached with rainwater flowing downwards the column, yielding 0–0.6 % of metal release [41]. This marginal metal release responded to the small rainwater flow rate (1–2 mL/min) that passed through the column, which was the opposite of what occurred in the field where abundant rainwater flux fell over the treated soil. (**2**) MCNPs manufactured on-site were large (76.6 nm) and formed chain-like aggregates. The growth of such nanoparticles resulted from the interaction between unwanted rainwater elements (see Table S1) and commercial-grade chemicals during their preparation. Many MCNPs had transport restrictions when traveling through the micropores of clay soils (1–100 nm) [93]. Hence, the nanoparticles accumulated on the soil surface, preventing their interaction with the subsurface soil cadmium. Former researchers reported that zero-valent iron nanoparticles (nZVI) having 18.1 ± 2.5 nm were transportable through saturated porous media (coarse and fine glass beads, clean sand, and sandy soil). Nonetheless, nanoparticles of larger size could not permeate the media and stayed on the top of the soil under treatment [98,99]. (**3**) native heterogeneous soils hindered the infiltration of the MCNPs because they contain 88 % of fines (clay) and cocoa litter under decomposition on the surface [78], so there was the same limitation of not having sufficient nanoparticles to react with the available cadmium; (**4**) the nanoparticles' size changes while transporting them to the injection wells. Iron salts in the MCNP synthesis enable the development of magnetic activity, leading to its aggregation by van der Waals and magnetic forces; thus, a stabilizing coverage is needed [100,101]. We intentionally omitted using polymeric materials as stabilizing agents in the MCNPs' preparation to prevent their aggregation over time. Because the more straightforward the procedure for fabricating MCNPs in the field is, the more accessible it is for the farmers to handle. However, MCPN formed aggregates with sizes of 166 ± 111 nm at the outlet of the feeding droppers during the field tests. The large aggregates, once deposited at the top of the well, could not be transported downwards of soil; instead, they flooded the injection wells and accordingly had fewer interactions with cadmium. (**5)** low cadmium concentrations in the soil of the Santa Lucía farm (0.45 mg Cd/kg) made the interaction with the MCNPs more random. The less cadmium is available in the soil, the less contact between the metal and the injected nanoparticles, reducing the Cd immobilization [81].

## 5 Conclusion

The field prototype for large-scale multicomponent nanoparticle fabrication is evidence of the practicality of this research. It includes a reactor tank, a mixing tank, a water collector vessel, and necessary accessories. The key feature is the design and build-up of a 0.5 m^3^ stainless steel reactor equipped with an impeller-blade agitation mechanism to keep the rotational velocity. Its performance, thoroughly investigated by CFD simulations, shows that after 60 s of agitation, the mixture is wholly homogenized inside the reactor, guaranteeing the reactions between chemicals and speeding up the formation of nanoparticles. However, the MCNPs prepared in the field prototype were large, 76.6 nm on average, and yielded chain-like aggregates of spherical MCNPs. The nanoparticles prepared on-site contain Fe^0^ in the core and FeS in the shell, as provided by EDX. It reveals the presence of an iron content of 90.7 % and sulfur of 1.81 %, unequivocally confirmed by peaks of the XRD spectrum at 2θ: 44.79°, 65.11° and 82° for elemental iron and at 2θ: 30° and 35.8° for pyrite (FeS).

Regarding the treatments, MCNPs injected in fixed-bed columns packed with screened clayey soils immobilized ∼80 % of available cadmium. However, in 5 m^2^ plots of the Santa Lucia farm soils dosed with MCNPs at a flow rate of 835 mL/min, only 22 % of available cadmium was immobilized. The discrepancy in the cadmium immobilization is attributed to the large size of MCNPs, heterogeneity of naturally treated soil, the even larger size of the nanoparticles at the outlet of the feeding droppers, the dissolution of solid phases due to abundant rainfall that fell in the study area during the field tests [97], and the low Cd concentration in the untreated soil.

On the other hand, 31 and 19 kg of cocoa beans CCN51 and Fino de Aroma, treated with 100 mL of nanoparticles/kg during the on-site beans' fermentation, yielded a metal removal of ∼20 %. Nevertheless, mixing ∼100 g of cocoa beans with 3 mL of MCNPs in a laboratory fermenter produced a cadmium removal of 80 % for Fino de Aroma and 69 % for CCN51. The difference in Cd removal between treatments can be attributed to the large size of MCNPs fabricated in the field, as they cannot diffuse well through the beans' 22.6 nm average pore diameter [91]. Also, the low cadmium concentration in cocoa beans from the Santa Lucia farm before treatment was an important issue that needed to be analyzed in determining the low metal removal. Nonetheless, the quality of the fermented cocoa beans treated with MCNPs produced seed index values, humidity content, and pH values that exceeded the local standards. Moreover, 91.33 % and 93.33 % of good fermentation resulted in the Fino de Aroma and CCN51 varieties above the 75 % standard of good fermentation [80].

Ultimately, our research holds potential for treating cocoa-producing soils, particularly those with higher cadmium concentrations. In such conditions, the Cd content in the available form would be higher, making it more susceptible to interacting with the MCNPs. This insight opens new avenues for applying this treatment in regions with higher cadmium concentrations, expanding our research scope and bringing realistic expectations to our farmers.

## CRediT authorship contribution statement

**Ambar Oñate:** Writing – original draft, Methodology, Investigation. **Carina Staël:** Project administration, Methodology, Investigation, Formal analysis, Conceptualization. **Darío Bolaños-Guerrón:** Writing – review & editing, Project administration, Investigation, Data curation, Conceptualization. **Carlos Pozo:** Methodology, Investigation, Conceptualization. **Daniela Vera:** Methodology, Investigation, Formal analysis. **Elena Torres:** Methodology, Investigation. **Carlos Naranjo:** Methodology, Investigation, Conceptualization. **Miguel Perugachi:** Methodology, Investigation. **Andrés Loayza:** Methodology, Investigation. **Joffe Pincay:** Investigation. **Manuel Carrillo:** Methodology, Investigation, Conceptualization. **Luis Cumbal:** Writing – review & editing, Writing – original draft, Project administration, Investigation, Funding acquisition, Formal analysis, Data curation, Conceptualization.

## Data availability statement

Data included in the article/supplementary material/referenced in the article.

## Funding

This study was funded by Secretaria Nacional de Educaciòn Superior Ciencia y Tecnologìa (10.13039/501100004299SENESCYT) of Ecuador through the project “Desarrollo y aplicaciòn de nanomaterials para la recuperaciòn de suelos contaminados con cadmio” Grant PIC-18-INE-ESPE-004.

## Declaration of competing interest

The authors declare that they have no known competing financial interests or personal relationships that could have appeared to influence the work reported in this paper.
