# Supplementary material for: Nanoparticles for treatment of cadmium-contaminated cocoa-growing soils and beans: Performance on metal immobilization and removal
Source: Heliyon. 2024 Dec 2;11(4):e40519. doi: 10.1016/j.heliyon.2024.e40519 (PMC11889560; doi:10.1016/j.heliyon.2024.e40519)
Supplement: Multimedia component 1 [file mmc1.docx]

**Supplementary material**


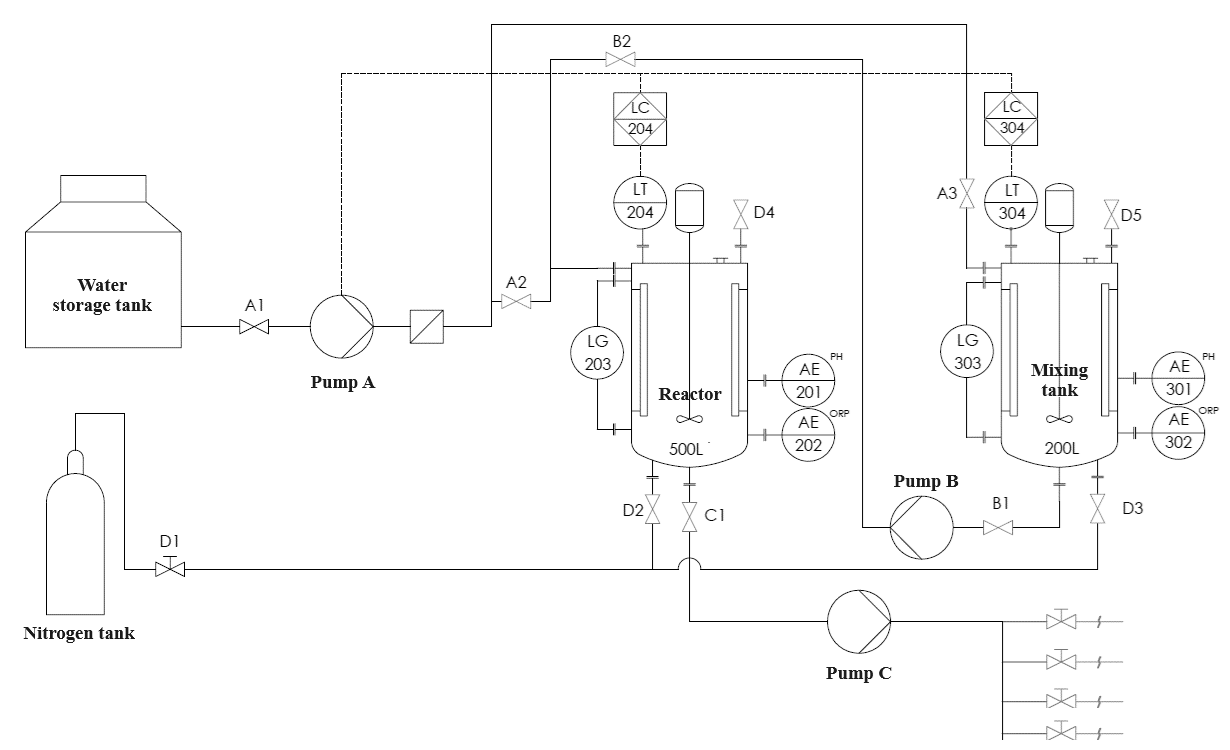


**Figure S1.** Flow diagram of the field prototype


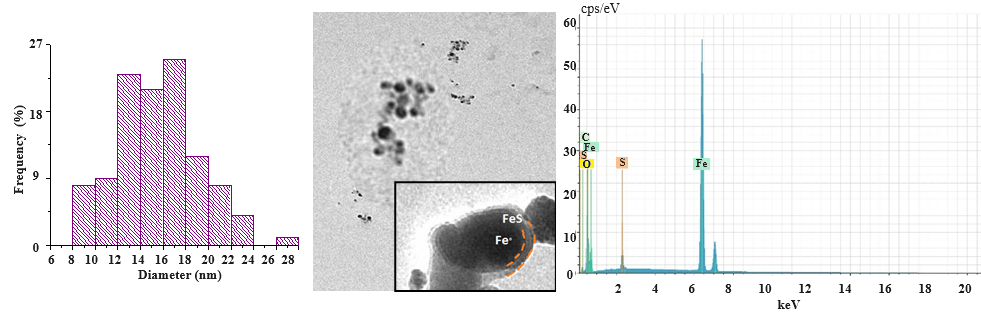


**A**

**C**

**B**

**A**

**Figure S2.** (A) Size, (B) morphology, and (C) chemical distribution of the MCNPs prepared in the laboratory.


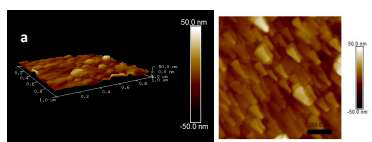


**B**

**A**

**Figure S3**. a) AFM image of the MCNPs prepared in the field using 591 mg/L Na_2_S.9H_2_O. b) Closeup of a section of the morphology

**Figure S4**. SEM scanning and mapping of iron (green) and sulfur (red) in a sample of MCNPs prepared in the field.

**Figure S5.** XRD spectrum of MCNPs prepared in the field.

**
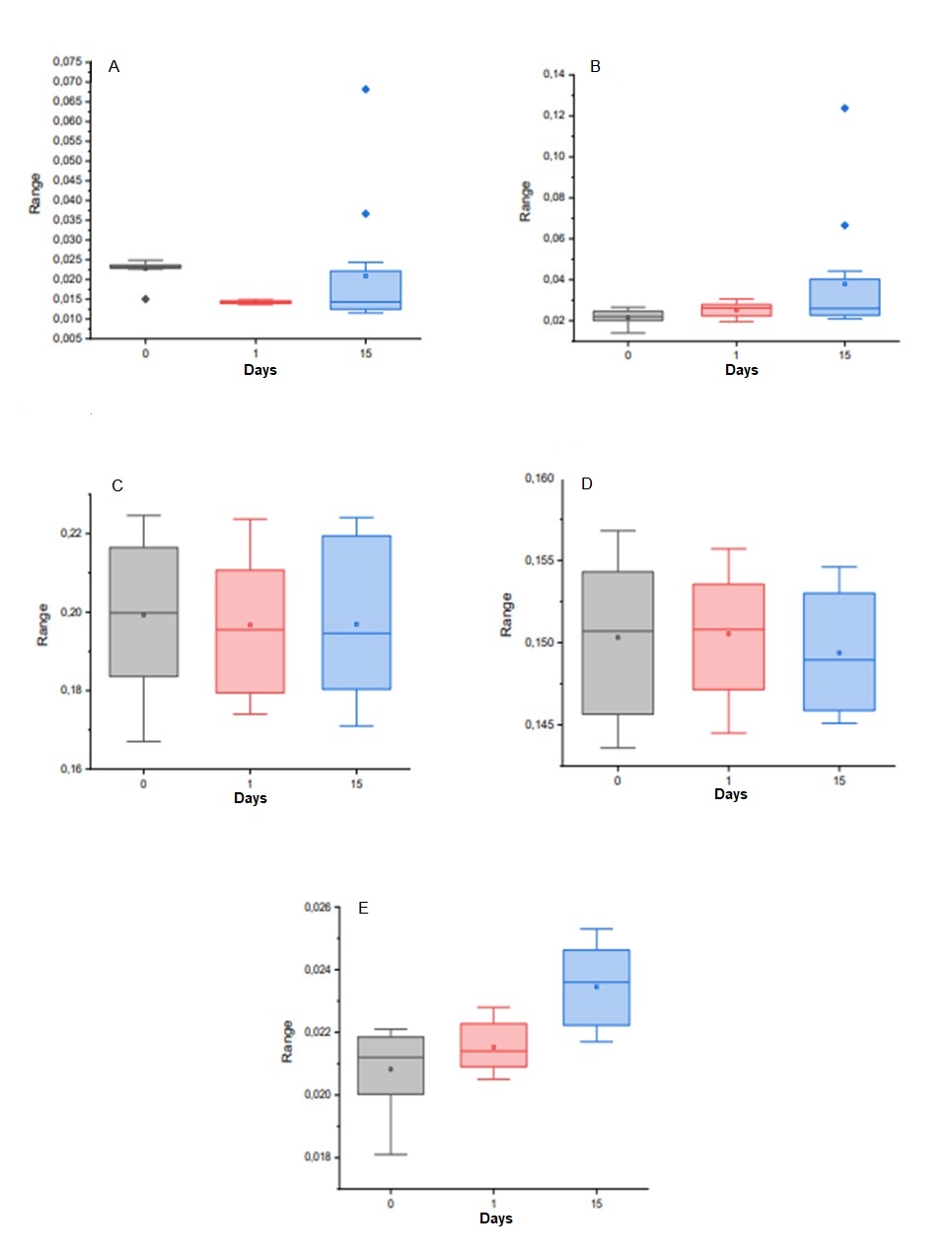
**

**Figure S6.**  Means of cadmium concentration in the five soil fractions before and after treatment with MCNPs. A) Exchangeable fraction (F1), B) Bound to carbonate fraction (F2), C) Oxidable fraction (F3), D) Reducable fraction (F4), and E) Residual fraction (F5).

**Table S1**. Physicochemical properties of water sources for the MCNPs fabrication using the field prototype

| Water Source | Electrical conductivity (mS/cm) | TDS (mg/L) | pH | DO  (mg/L) | Main ions (mg/L) | Performance for MCNPs fabrication* |  |
| --- | --- | --- | --- | --- | --- | --- | --- |
|  |  |  |  |  |  |  |  |
| INIAP Groundwater | 192.9 | 125 | 8.09 | 5.76 | Na^+^ = 20 Ca^2+^ = 12  Mg^2+^ = 4  Cl^–^ = 40 HCO_3_^–^ = 30  NO_3_^–^ = 5 SO_4_^2–^ = 2 NO_2_^–^ = 2 PO_4_^3–^ = 2 | Poor |  |
| ESPE Groundwater | 600 | 386 | 8.05 | 6.51 | Na^+^ = 40 Ca^2+^ = 50  Mg^2+^ = 20  Cl^–^ = 130 HCO_3_^–^ = 80  NO_3_^–^ = 25 SO_4_^2–^ = 10 NO_2_^–^ = 10 PO_4_^3–^ = 5 | Poor |  |
| Santa Lucia farm rainwater | 92.7 | 60 | 6.88 | 4.39 | Na^+^ = 8.5 Ca^2+^ = 2.2  Mg^2+^ = 4.0  Cd^2+^ = 0.007  Cl^–^ = 5.4 HCO_3_^–^ = 24.0 NO_3_^–^ = 1.5 SO_4_^2–^ = 1.8 | Good |  |
|  |  |  |  |  |  |  |  |
|  |  |  |  |  |  |  |  |

- **Poor**: No zero-valent nanoparticles formed because the high chloride content in two groundwaters precluded the reduction of the Fe^3+^ to Fe^0^ and further being covered with the FeS tiny film.
- **Good**: Zero-valent nanoparticles were successfully formed. The chloride in the rainwater was low, thus allowing the reduction of the Fe^3+^ to Fe^0^ and the coverage of the nano FeS film.

**Table S2.** ANOVA for cadmium immobilization in S001 soil after treatment dosing with NPs, Fraction 1

| Variable | Df | Sum Sq | Mean Sq | F value | Pr(>F) |
| --- | --- | --- | --- | --- | --- |
| Model | 2.97 | 5 | 0.59 | 6918.77 | <0.0001 |
| Treatment | 2.97 | 5 | 0.59 | 6918.77 | <0.0001 |
| Error | 0.00041 | 48 | 0.000086 |  |  |
| Total | 2.98 | 53 |  |  |  |

**Table S3.** ANOVA for cadmium immobilization in S002 soil after treatment dosing with NPs, Fraction 1

| Variable | Df | Sum Sq | Mean Sq | F value | Pr(>F) |
| --- | --- | --- | --- | --- | --- |
| Model | 2.95 | 5 | 0.59 | 4733.76 | <0.0001 |
| Treatment | 2.95 | 5 | 0.59 | 4733.76 | <0.0001 |
| Error | 0.01 | 48 | 0.00012 |  |  |
| Total | 2.95 | 53 |  |  |  |

**Table S4.** ANOVA for cadmium removal from the Fino de Aroma beans after fermentation with doses of NPs

| Variable | Df | Sum Sq | Mean Sq | F value | Pr(>F) |  |
| --- | --- | --- | --- | --- | --- | --- |
| A: NPs Type | 1 | 152.3 | 152.3 | 2.955 | 0.111253 |  |
| B: NPs Volume | 2 | 1686.3 | 843.1 | 16.367 | 0.000373 |  |
| AB | 2 | 97.3 | 48.6 | 0.944 | 0.416066 |  |
| Error | 12 | 618.2 | 51.5 |  |  |  |

**Table S5.** ANOVA for cadmium removal from the CCN51 beans after fermentation with doses of NPs

| Variable | Df | Sum Sq | Mean Sq | F value | Pr(>F) |  |
| --- | --- | --- | --- | --- | --- | --- |
| A: NPs Type | 1 | 276.9 | 276.9 | 1.271 | 0.282 |  |
| B: NPs Volume | 2 | 2929.9 | 1465.0 | 6.725 | 0.011 |  |
| AB | 2 | 72.1 | 36.1 | 0.166 | 0.849 |  |
| Error | 12 | 2613.9 | 217.8 |  |  |  |
